# Design of combined stationary and mobile battery energy storage systems

**DOI:** 10.1371/journal.pone.0260547

**Published:** 2021-12-01

**Authors:** Hassan S. Hayajneh, Maximiliano Lainfiesta Herrera, Xuewei Zhang

**Affiliations:** 1 Texas A&M University-Kingsville, Kingsville, TX, United States of America; 2 Rocky Mountain Institute, Boulder, CO, United States of America; Northeast Electric Power University, CHINA

## Abstract

To minimize the curtailment of renewable generation and incentivize grid-scale energy storage deployment, a concept of combining stationary and mobile applications of battery energy storage systems built within renewable energy farms is proposed. A simulation-based optimization model is developed to obtain the optimal design parameters such as battery capacity and power ratings by solving a multi-objective optimization problem that aims to maximize the economic profitability, the energy provided for transportation electrification, the demand peak shaving, and the renewable energy utilized. Two applications considered for the stationary energy storage systems are the end-consumer arbitrage and frequency regulation, while the mobile application envisions a scenario of a grid-independent battery-powered electric vehicle charging station network. The charging stations receive supplies from the energy storage system that absorbs renewable energy, contributing to a sustained DC demand that helps with revenues. Representative results are presented for two operation modes and different sets of weights assigned to the objectives. Substantial improvement in the profitability of combined applications over single stationary applications is shown. Pareto frontier of a reduced dimensional problem is obtained to show the trade-off between design objectives. This work could pave the road for future implementations of the new form of energy storage systems.

## 1. Introduction

**Fig 1:**
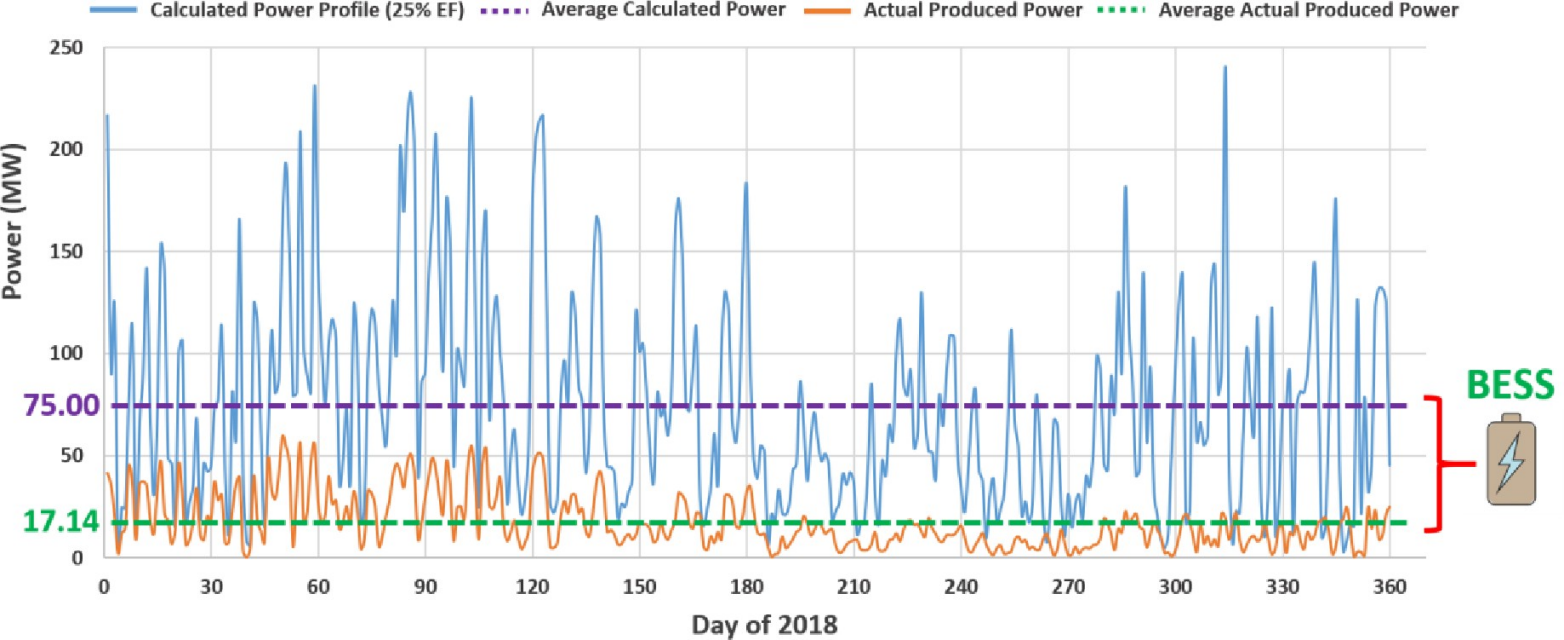
Comparison between actual produced power profile of 250 MW wind farm in Texas, USA in 2018 and calculated power profile assuming 25% efficiency factor for wind turbines.

Battery energy storage systems (BESSs) have been deployed to meet the challenges from the variability and intermittency of the power generation from renewable energy sources (RESs) [1–4]. Without BESS, the utility grid (UG) operator would have to significantly curtail renewable energy generation to maintain system reliability and stability [5,6]. In the case of wind energy, examples of non-fully utilized wind farms are provided in [7–9]. Fig 1 compares the actual grid power injection from a 250 MW wind farm (Chapman Ranch, Texas) in 2018 with the calculated power generation capability. The wind farm averaged an actual power production of about 17.14 MW (~7% of its capacity). Using the weather conditions in 2018 for the same area (acquired from [10]), and assuming the wind turbines with a 25% efficiency factor (EF), the calculated power production without curtailment could have been 3~4 times higher than the actual power injection. The difference between the two profiles forms the potential wasted power (average to average) that could have been stored in energy storage systems for other applications.

Grid-scale, stationary BESSs have had multiple conventional applications such as (i) end-consumer arbitrage (ECA) [11,12] to enable consumers to take advantage of lower energy prices due to BESS, (ii) resource adequacy and reserve capacity [13–15] ensuring the safe and reliable operation of the UG in real-time by providing sufficient resources, (iii) frequency regulation (FR) [16,17] to maintain the AC frequency within tight tolerance bounds and increase the grid stability, (iv) voltage support [18] to absorb or deliver reactive power and serve to keep a specific voltage level on the grid, (v) black-start [19,20] to restore power plant’s electricity or portions of electric grid to operation after a total or partial shutdown, and (vi) transmission congestion relief [21,22] to enhance the transmission network capacity when the capability of demand for transmission is surpassed. BESSs have gained increasing popularity in the latest decades. According to the U.S. Energy Information Administration (EIA), at the beginning of 2018, the large-scale battery storage in the U.S. was accounted for 708 MW of powered capacity and almost 900 MWh of energy capacity [23]. Worldwide, the installed capacity of BESS is predicted to rise by nearly 20 GW per year [24]. However, the main bottleneck limiting the BESS deployment remains the fairly high costs of the storage technology [25].

In two previous studies [7,26], driven by data records which show that a wind farm supplies energy to the UG at levels much lower than its real-time capacity (Fig 1), and inspired by [27] which demonstrated improvements of the investment attractiveness for stationary BESSs by combining applications, it was proposed that the BESS profitability can be significantly enhanced if part of it can be deployed as mobile energy sources to provide backup power to critical facilities or for emergencies, to deliver continuous power supply during grid maintenance and repair, and more interestingly, to meet the energy demand of electric vehicle charging station (EVCS) networks.

### 1.1 The motivation for the grid-independent BESS

The transportation sector witnessed a dramatic growth in the share of electric vehicles (EVs) which in return helps to mitigate the disastrous effects of greenhouse gas (GHG) emissions on the planet, however, this growth will also cause serious issues (e.g., power congestion) to the UG when about one-sixth of the vehicles on the road go electric over the next decade [28,29].

**Fig 2:**
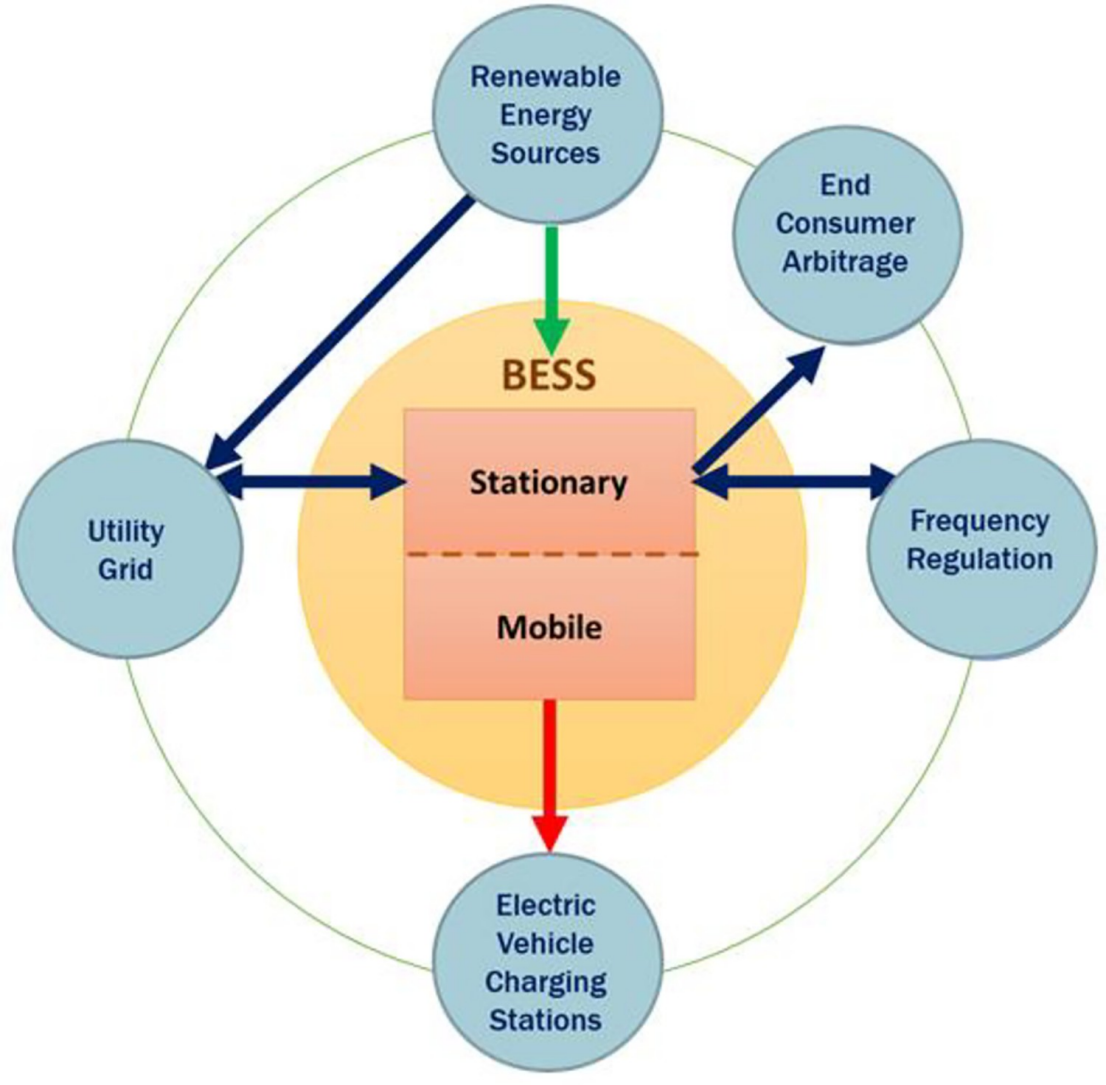
Diagram of the proposed BESS.

**Fig 3:**
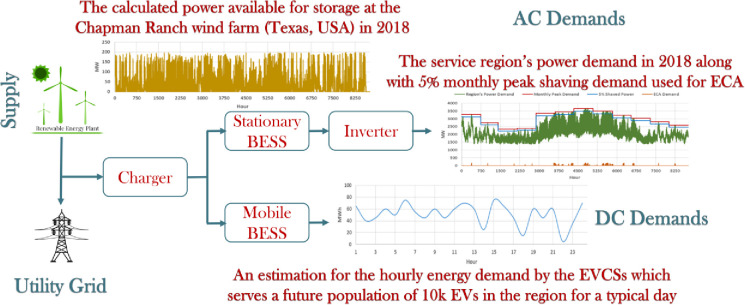
Diagram of the conceptual BESS model with the proposed applications. Better resolutions for the embedded figures are available in Fig 4.

As an alternative solution to the reinforcement of the electric grid, and opposite to keep connecting the new energy storage systems to the utility grid as in [30,31], one may consider the deployment of grid-independent battery-powered EVCS network designs, with the large-scale batteries (MWh) shipped back and forth between EVCSs and BESS plant by fleets of electric truck. From the BESS plant (supplier) point of view, this would account for a continuous DC demand, in contrast to the grid services (AC demand) that are generally intermittent and temporary. With more battery utilization and a guaranteed higher revenue in return, the mobile BESS concept (as illustrated in Fig 2) holds the promise to become a sustainable business model. The proposed BESS stores the extra available power from the RES farms and uses it in several applications: stationary applications like end-consumer arbitrage and frequency regulation (AC demands), and mobile application (DC demand, here meeting demands of the electric vehicle charging stations). The arrows indicate the directions of flow of energy. The conceptual BESS model and the proposed applications are shown in Fig 3.

There are two limitations of [7]: firstly, due to the unavailability of real data, algorithms were used to generate time series of load and portions of wind power production; secondly, only two BESSs with specific capacities were considered, i.e., no optimal design. To address these problems, this work develops a data-driven, simulation-based optimization model to enlighten the planning of a BESS next to RES such as a wind farm. The model can generate key design parameters such as the capacity and power rating by solving a multi-objective optimization problem that aims to maximize the economic profitability index (PI), the energy provided into transportation electrification, the load peak shaving, and the amount of wind energy stored. We consider two operation modes (DC priority and AC priority) and different sets of weights assigned to the above objectives. The results confirm the substantial increase in the profitability of the combined stationary and mobile applications over only stationary applications. This work is intended to help real-world scenarios and future demonstration projects.

The rest of the paper is organized as follows. In Section II, the system configuration, data sources, and model formulation are described. The main results and discussions are presented in Section III. Some conclusions and suggestions for future work can be found in Section IV.

## 2 Problem description

Opposite to the available small-scale technologies of BESS in supporting the electric vehicles charging stations such as battery swapping [32–35], hydrogen storage [36,37], and fuel cells [38,39], in this work we propose a new framework of large-scale BESS (each battery unit is assumed to be within a capacity of 5 MWh). The available technologies are not found to be very popular at large scale, in addition to their reliance on the utility grid as the primary feeder of energy to the EVCSs. Part of the BESS we are proposing here is movable (mobile) and used to power grid-independent EVCS networks, as shown in Figs 2 and 3. The remaining part of the BESS is for conventional, stationary applications, i.e., ancillary services to the UG. The focus is to develop a data-driven, simulation-based optimization framework to obtain the optimal parameters of the proposed BESS. In this section, the data inputs of the model will be described first, then the modules of the simulation model will be introduced, and finally, some technical details of the optimization process will be provided.

### 2.1 Data inputs

**Fig 4:**
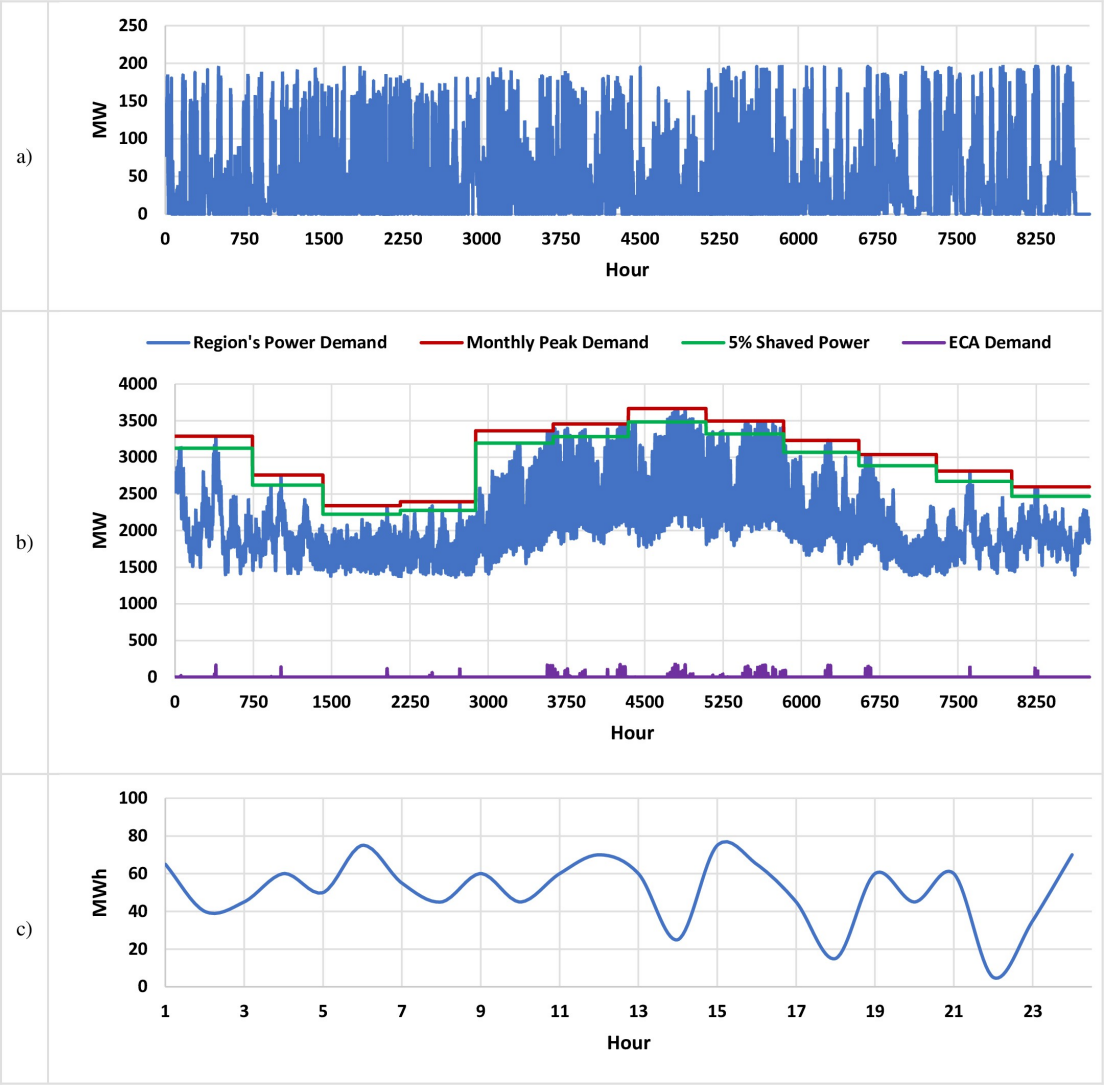
The simulation model’s data inputs. a) The calculated curtailed power available for storage at the Chapman Ranch wind farm (Texas, USA) during 2018 after injecting power to the UG, with the real-time generation estimated based on actual weather data in the region. b) The region’s load demand in 2018 along with 5% monthly peak shaving used for ECA. c) An estimation for the hourly energy demand by the EVCSs which serves a future population of 10k EVs in the region for a typical day [42].

The data is collected from an operating RES, the Chapman Ranch wind farm (Texas, USA) with an installed generation capacity of 250 MW, including the actual hourly weather data (wind speed, air density, etc.) for the same region [10]. Using the same method used in [7], the hourly profile of the real-time wind power generation capacity in 2018 is calculated. Then deducting the actual power injected into the utility grid from the wind farm (data source: ERCOT) to estimate the amount of energy available for storage, which is plotted in Fig 4(A). This is the upper limit of the wasted power (assuming all wind turbines operational) that would not be injected into the UG if there is no BESS. One objective of the BESS design in this work is to maximize the “absorption” of the extra power from the wind farm, thus maximizing the utilization of the RES.

Three BESS applications are considered in this work; two are provided by the stationary portion of the BESS. In [27], among the conventional, stationary applications of BESS, it was shown that, in the scenarios under study, a combination of end-consumer arbitrage as the primary application and frequency regulation as the secondary application results in the highest profitability index. The present work also considers these two applications as the AC applications (stationary), in addition to the new DC application (mobile).

The first stationary BESS application considered in this study is end-consumer arbitrage, which creates value for potential industrial consumers who may also invest in the project. When the real-time demand of a consumer approaches the monthly peak (*D*_*peak*_(*m*)), the BESS will supply power to shave the peak demands and reduce the demand charges from the utilities. According to the wind farm service region’s load profile in 2018 (Fig 4(B), blue curve), and assuming the peak shaving factor is 5% (i.e., cutting the monthly peak by 5%), the profile of the BESS’s demand due to ECA application can be calculated, as shown in Fig 4(B) (purple curve).

The other stationary application of BESS is frequency regulation (FR). The annual revenue generated from the frequency regulation application is anticipated as the product of FR capacity reserved in the BESS (in MW) and the service price (in $/MW-year [40,41]). Since the available FR event record lacks details and individual FR events are extremely brief and for a short amount of time (10 s ~ 1 min), it is excluded from the simulation model which has a time step of 1 hour. Instead, the model treats the FR capacity as a reserved portion of the power ratings of BESS charger/inverter and sets upper and lower bounds of the state-of-charge (SoC) of the BESS that correspond to capacity reserved for FR.

The third application will be met by the mobile portion of the BESS. The demand profile plotted in Fig 4(C) is used. Note that in a previous work [42], for an EVCS network that serves a population of 10,000 EVs, the total daily demand is estimated to be ~1200 MWh. A random vector with a mean of 50 MWh is generated as the hourly demand profile *D*_*EVCS*_(*h*). While a more realistic EVCS demand profile can change the numerical details, it would not change the main conclusion that the introduction of the mobile application can improve both the utilization of renewables and the profitability index.

### 2.2 Simulation modeling

**Fig 5:**
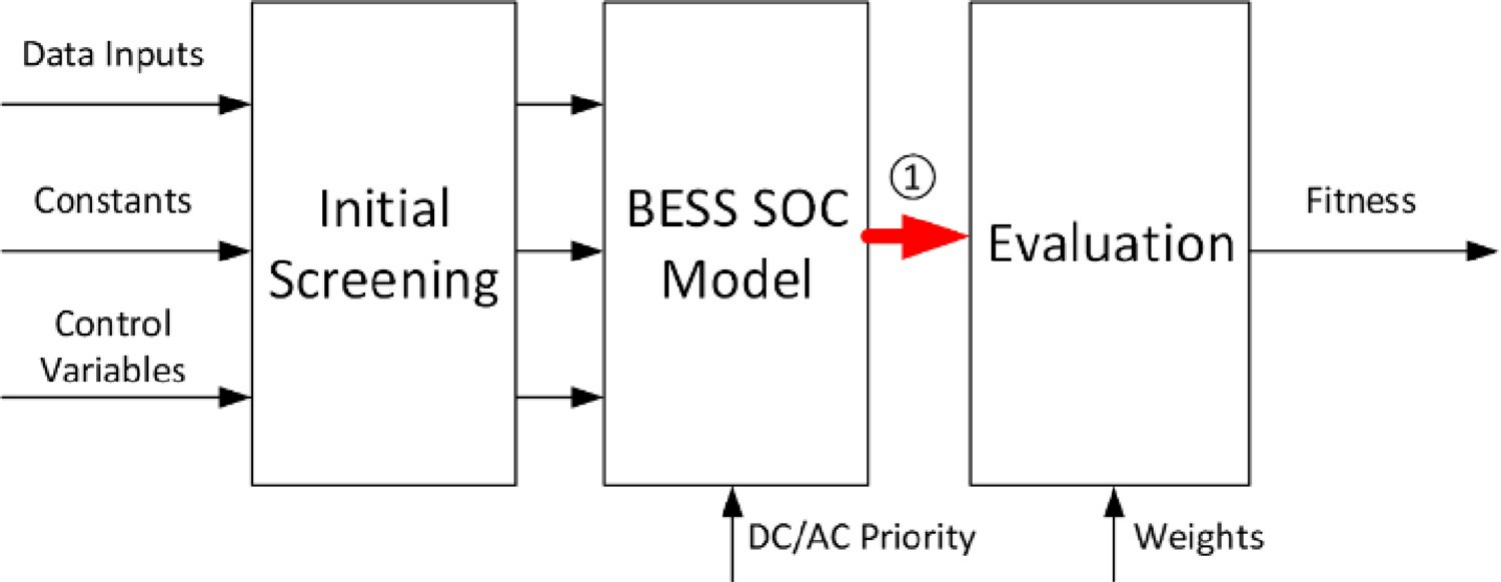
Block diagram representing the simulation model in the User Mode. The red arrow is explained in the text.

The simulation model can be run in two modes: The User Mode and the Designer Mode. The User Mode can evaluate the performance indicator (or fitness) of a given BESS, while the Designer Mode can search for the optimal set of parameters that correspond to the highest fitness. Fig 5 presents the block diagram of the simulation model in the User Mode. The model inputs are the data shown in Section II.A, some constants (including physical/financial parameters that are fixed in the simulations, listed in Table 1), and the control variables the values of which are specified by the users. As listed in Table 2, the model has six control variables (CV) along with their limits. The bounds for all control variables are obtained from the initial solution of optimization process in addition to an assumed optimization safety margin.

**Table 1:** Model constants and their values used in this study.

Symbol	Meaning and Units	Value
*dc*	The demand charges ($/MW)	10.3k [27]
*pt*	Power tariff set by the UG ($/MWh)	50
*η*_*charger*_	Efficiency of the Charger (%)–used when calculating the power absorbed from RES into BESS	95 [43]
*η*_*inverter*_	Efficiency of the Inverter (%)–used when calculating the power provided into AC applications	95 [43]
*WACC*	Weighted Annual Cost of Capital (%)	5.25 [27]
*DC*_*rate*_	The price for energy provided to EVCSs ($/MWh) [Assumed]	100
*B*_*cost*_	The cost of the Battery ($/MWh)	100k
*C*_*cost*_	The cost of the Charger ($/MW)	80k
*I*_*cost*_	The cost of the Inverter ($/MW)	80k
*B*_*OMfactor*_	Annual cost of battery operation & maintenance as percentage of *B*_*cost*_ (%) [Assumed]	5
*C*_*OMfactor*_	Annual cost of charger operation & maintenance as percentage of *C*_*cost*_ (%) [Assumed]	5
*I*_*OMfactor*_	Annual cost of inverter operation & maintenance as percentage of *I*_*cost*_ (%) [Assumed]	5
*FR*_*rate*_	Frequency regulation price ($/MW-yr). Based on ERCOT 10.25% reserve margin [44]	100k
*PL*	Project life time (Years)	20

The first module of the model shown in Fig 5 is called the Initial Screening, which examines the compatibility within the model inputs and the feasibility of other parameter settings. Among the screening criteria,*CV*_2_ should be lower than *CV*_1_; *CV*_5_ should never exceed the value of *CV*_3_ or *CV*_4_; along with other constraints to be detailed below. As a model assumption, the reserved SoC for FR in the BESS is equal to the FR reserved power multiplying by *δ* = 1/6 hour, which is mandated to be below half of the size of the stationary portion of the BESS (*CV*_1_−*CV*_2_). Note that this module will be disabled during the optimization process in the Designer Mode since the criteria are included as constraints there; Initial Screening is unnecessary.

**Table 2:** Control variables and their bounds.

CV	Symbol	Meaning and Units	Lower bound	Upper bound
*CV*_1_	*BESS*_*size*_	The total size of the BESS (MWh)	350	2000
*CV*_2_	*BESS*_*mobile*_	The size of the mobile portion of the BESS (MWh)	50	500
*CV*_3_	*P*_*charger*_	The power rating of the charger (MW)	250	600
*CV*_4_	*P*_*inverter*_	The power rating of the inverter (MW)	250	600
*CV*_5_	*FR*_*reserve*_	The power reserved for frequency regulation (FR) application (MW)	10	100
*CV*_6_	*SF*	The shaving factor of peak demand (%)	0.5	15

**Fig 6:**
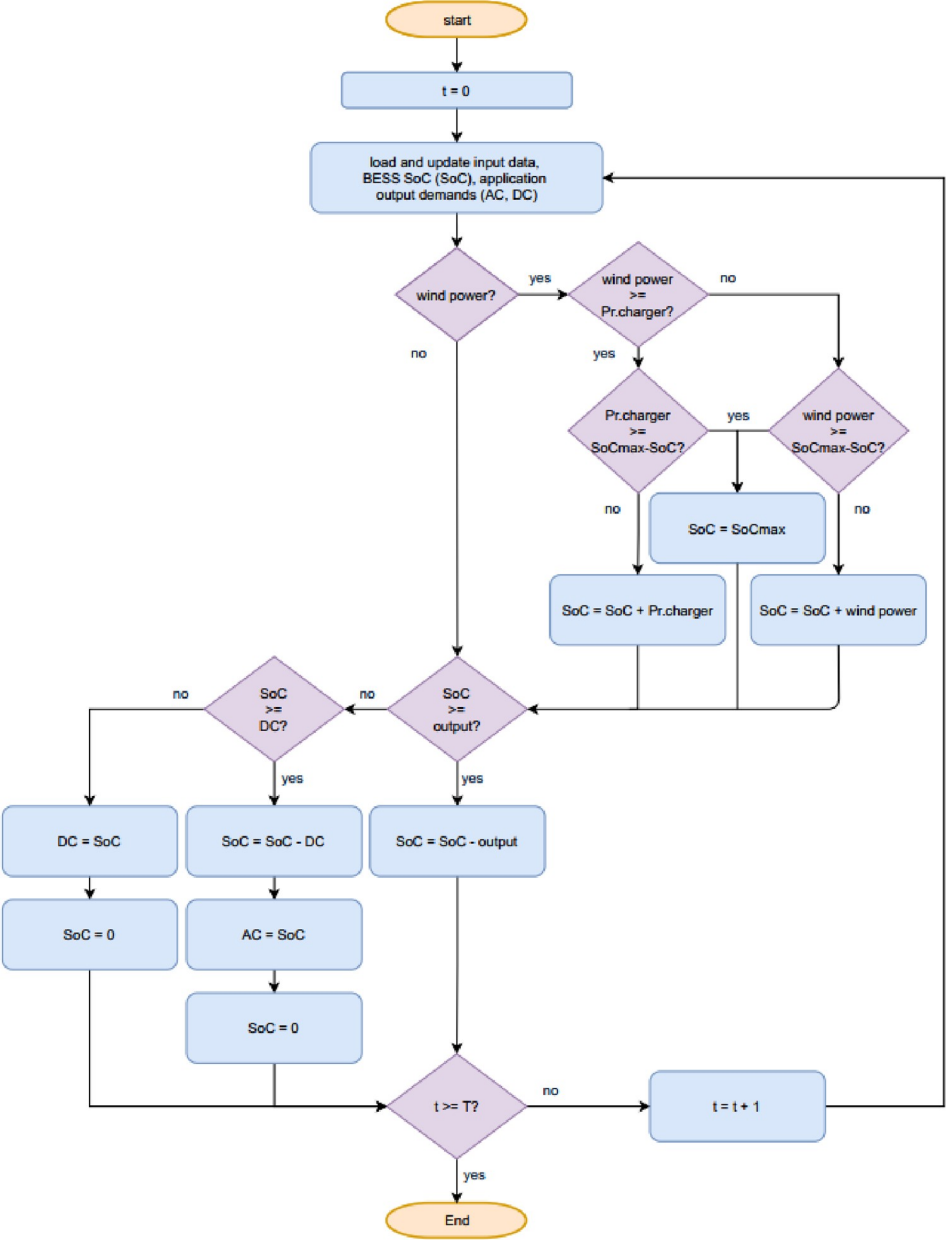
The flowchart of supplying all demands while prioritizing the DC demands.

After the control variables pass the initial screening, all the inputs are passed on to the BESS SoC model. The BESS SoC model is simulated hour-by-hour for a year to make decisions on power absorption from the RES and how the demands are met. There are two operational modes of the proposed BESS, namely DC priority and AC priority. Here DC/AC priority means that if at the current hour, the available energy supply (wind farm extra and BESS combined) cannot meet 100% of the combined AC (stationary applications) and DC (mobile applications) demands, the system will try to meet the DC/AC demand first. The BESS SoC dynamics follows previous work [25]. Fig 6 presents the flowchart for the scenarios of supplying all demands, with more focus on the DC priority. There are lower and upper bounds of SoC as required by the FR reserve. Within this range, the SoC will be updated hourly by the difference between supply and demand. The only complication in the model is that the actual mobile portion size is time-dependent since every hour fully-charged batteries are shipped out to EVCSs and drained batteries are transported back by trucks dispatched out an hour earlier.

There are four hourly profiles as the output of the model: (a) *P*_*S*_, the power produced by the fully operational RES less the amount injected to the UG, (b) *P*_*A*_, the actual power absorbed by the BESS from the RES, (c) *P*_*ECA*_, the actual power supplied for peak shaving (ECA), and (d) *P*_*EVCS*_, the actual power provided for mobile applications (EVCSs). These data will be diverted to the Evaluation module to generate the BESS performance indices.

There are four normalized indices between 0 and 1, and the higher the value of each index, the better.

(*i*) Index 1 (*f*_1_), the investment attractiveness (IA): is defined as the ratio of the number of years the PI being above 1 to the project lifetime (20 years in this study). Conceptually, it includes the reduction of demand charges and the corresponding energy charge (ECA), FR revenue, and net profits from the mobile application of the BESS. For the latter, $100/MWh is assumed, after deduction of the transportation expenses (truck and labor) from the selling price. Higher values of IA mean more years with net profits. Therefore, the IA index reflects the financial viability of the proposed BESS design.

The calculation of the PI follows:

$${PI}_{y}={NPV}_{y}/C_{invest}$$ (1)

where *C*_*invest*_ = *B*_*cost*_*BESS*_*size*_+*C*_*cost*_*P*_*charger*_+*I*_*cost*_*P*_*charger*_ stands for the initial capital investment; and *NPV*_*y*_, the net present value ($) up to the end of year *y*, is calculated according to:

$${NPV}_{y}=\sum_{t=0}^{y}\frac{{CF}_{t}}{{\left(1+WACC\right)}^{t}}$$ (2)

where *t* represents the year in which *CF*_*t*_ is the cash flow ($) (the initial investment at *t* = 0 is set to be negative; for each subsequent year, the cash flow is the value generated minus the operating expenses), and *WACC* stands for the weighted average cost of capital. The annual cash flow (*CF*) that represents the difference between the monetary value generated (*VG*) by the three BESS applications and the operation and maintenance costs (*OM*_*cost*_) for a year is estimated as:

$$CF=VG-\sum_{B,C,I}{OM}_{cost}$$ (3)

where *VG* combines the annual monetary income generated by the energy supplied to EVCSs (*VG*_*EVCS*_), the capacity reserved for FR (*VG*_*FR*_), and the energy provided to the ECA (*VG*_*ECA*_):

$$VG={VG}_{EVCS}+{VG}_{FR}+{VG}_{ECA}$$ (4)

$${VG}_{EVCS}={DC}_{rate}\sum_{h=1}^{8760}P_{EVCS}(h)$$ (5)

$${VG}_{FR}={FR}_{reserve}{FR}_{rate}$$ (6)

$${VG}_{ECA}=dc\sum_{m=1}^{12}P_{shaved}(m)+pt\sum_{h=1}^{8760}P_{ECA}(h)$$ (7)

In Eq (7), *P*_*shaved*_ is each month’s actual shaved peak demand that can be obtained by the simulation. And *OM*_*cost*_ in Eq (3) consists of the contributions from the batteries (B), the chargers (C), and the inverters (I):

$$\sum_{B,C,I}{OM}_{cost}={B\_OM}_{cost}+{C\_OM}_{cost}+{I\_OM}_{cost}$$ (8)

$${B\_OM}_{cost}=B_{OMfactor}B_{cost}{BESS}_{size}$$ (9)

$${C\_OM}_{cost}=C_{OMfactor}C_{cost}P_{charger}$$ (10)

$${I\_OM}_{cost}=I_{OMfactor}I_{cost}P_{inverter}$$ (11)

(*ii*) Index 2 (*f*_2_): is the ratio of the actual mobile demand met and the total mobile demand according to the input data.

(*iii*) Index 3 (*f*_3_): is the ratio of the actual peak shaving demand met and the peak shaving demand corresponding to the targeted peak shaving factor. These performance indices (2&3) show the capability of the BESS to meet the stationary and mobile demands described above. Therefore, they will be specifically considered in the multi-objective optimization in the next section.

(*iv*) Index 4 (*f*_4_): is the ratio of the actual power from the RES stored by the BESS to the total power available for storage according to the input data. Index 4 shows the performance of the BESS in maximizing the utilization of renewables that would be otherwise wasted.

In summary, the calculation of the four indices follows:

$$f_{1}=\sum_{y=1}^{PL}I_{PI}(y)/PL$$ (12)

$$f_{2}=\underset{h}{\min}\left[P_{EVCS}(h)/D_{EVCS}(h)\right]$$ (13)

$$f_{3}=\underset{m}{\min}\left(P_{shaved}(m)/\left[SF∙D_{peak}(m)\right]\right)$$ (14)

$$f_{4}=\underset{h}{\min}\left[P_{A}(h)/P_{S}(h)\right]$$ (15)

where *I*_*PI*_(*y*) = 1 if *PI*_*y*_≥1 and 0 otherwise. In the cases of PI < 1 for all years (financial losses), *f*_1_ can be set to -1 as penalty. The hourly mobile demand *D*_*EVCS*_(*h*), monthly peak load *D*_*peak*_(*m*), and available energy for storage *P*_*S*_(*h*) are from the input data.

### 2.3 Optimization method

In the Designer Mode, the Genetic Algorithm (GA) is used as the solution method to find the optimal values of the control variables that maximize the performance indices in Eqs (12)–(15). The selection of the genetic algorithm came based on the following three advantageous points: (a) the Designer Mode represents a constraint problem (the six different constraints will be discussed next) where the GA is capable of solving such optimization problems, (b) the GA is powerful in repeatedly modifying the population of each individual solutions which in return benefits maximizing the performance indices, (c) based on random number generation, the GA obtains the next population by computation. The indices are evaluated at each iteration (i.e., each set of control variable values), which runs an hourly simulation of the system for a year. By choosing a vector of weights of the indices ***w*** = [*w*_1_, *w*_2_, *w*_3_, *w*_4_], the weighted average of the four indices defines a fitness function *F*, which is also an objective function in the Designer Mode. This work first optimizes *F* to validate the numerical algorithm and conduct preliminary analysis. Then a multi-objective (focusing on *f*_2_ and *f*_3_) optimization problem will be formulated and show the pareto frontier. The statement of the single objective optimization problem is:

$$Maximize\;F=\sum_{m=1}^{4}w_{m}f_{m}$$ (16)

Subject to:

$$\begin{array}{l} {CV}_{2}\geq \max\left(D_{EVCS}\right)\\ {CV}_{1}-{CV}_{2}-2\delta ∙{CV}_{5}\geq 0\\ {CV}_{1}-{CV}_{3}\geq 0\\ {CV}_{1}-{CV}_{4}\geq 0\\ {CV}_{3}-{CV}_{5}\geq \max\left(P_{S}\right)\\ {CV}_{4}-{CV}_{5}-\max\left(D_{peak}\right)∙({CV}_{6})\geq 0 \end{array}$$

Additionally, Table 2 provides the bounds for each control variable. The constraints combined are a necessary condition of the operational viability of the BESS.

**Fig 7:**
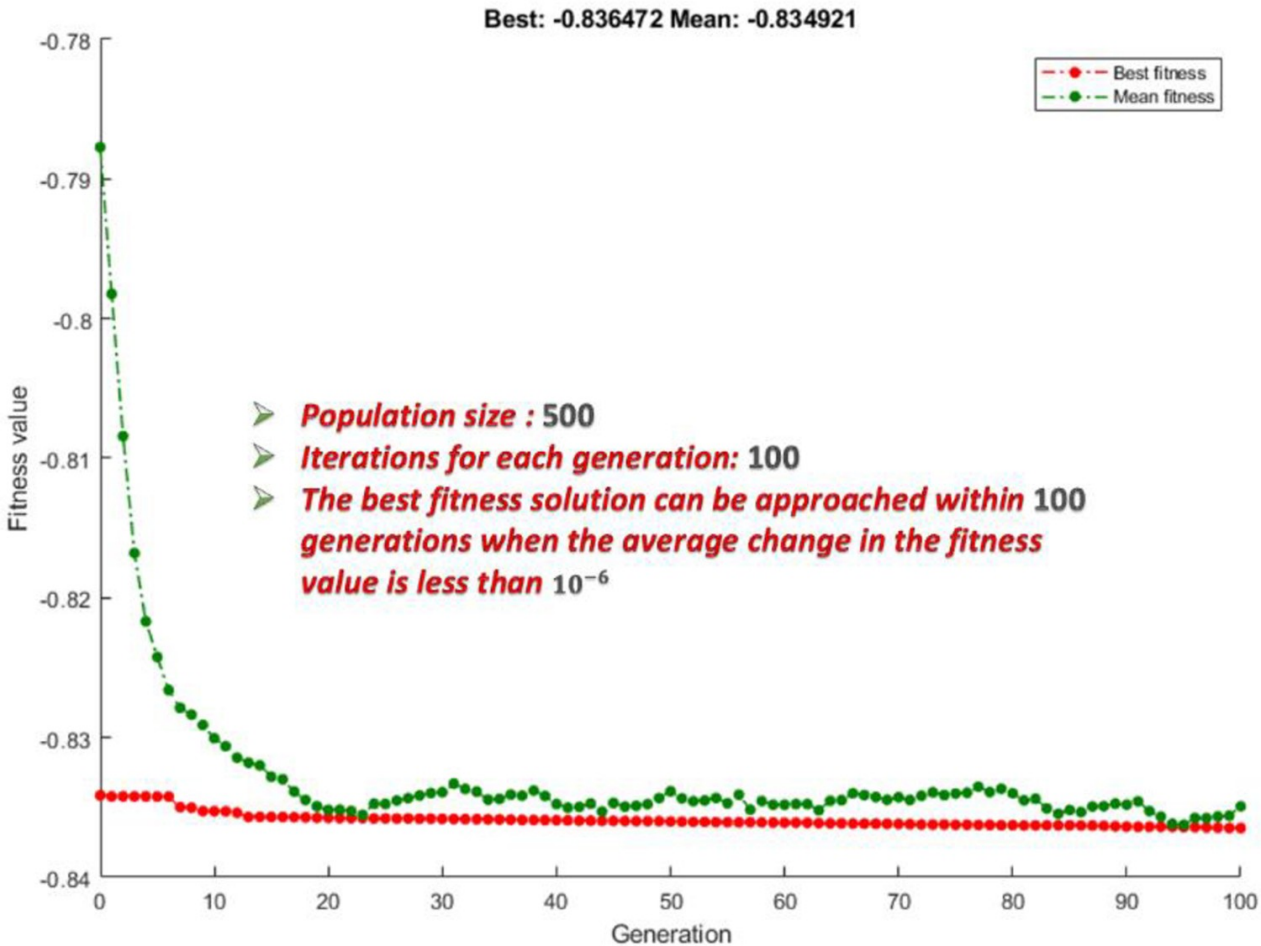
The convergence plot of GA for the equal weights’ scenario (*w*_*i*_ = 0.25) and DC priority. In general, the value for the best fitness can be attained within 100 generations.

The simulation model and the optimization processes are implemented in MATLAB. The GA settings are a population size of 500 and 100 iterations for each generation. Fig 7 shows a sample of convergence plot for equal weights.

## 3. Results and discussion

### 3.1 Optimization of the weighted average of four indices

**Fig 8:**
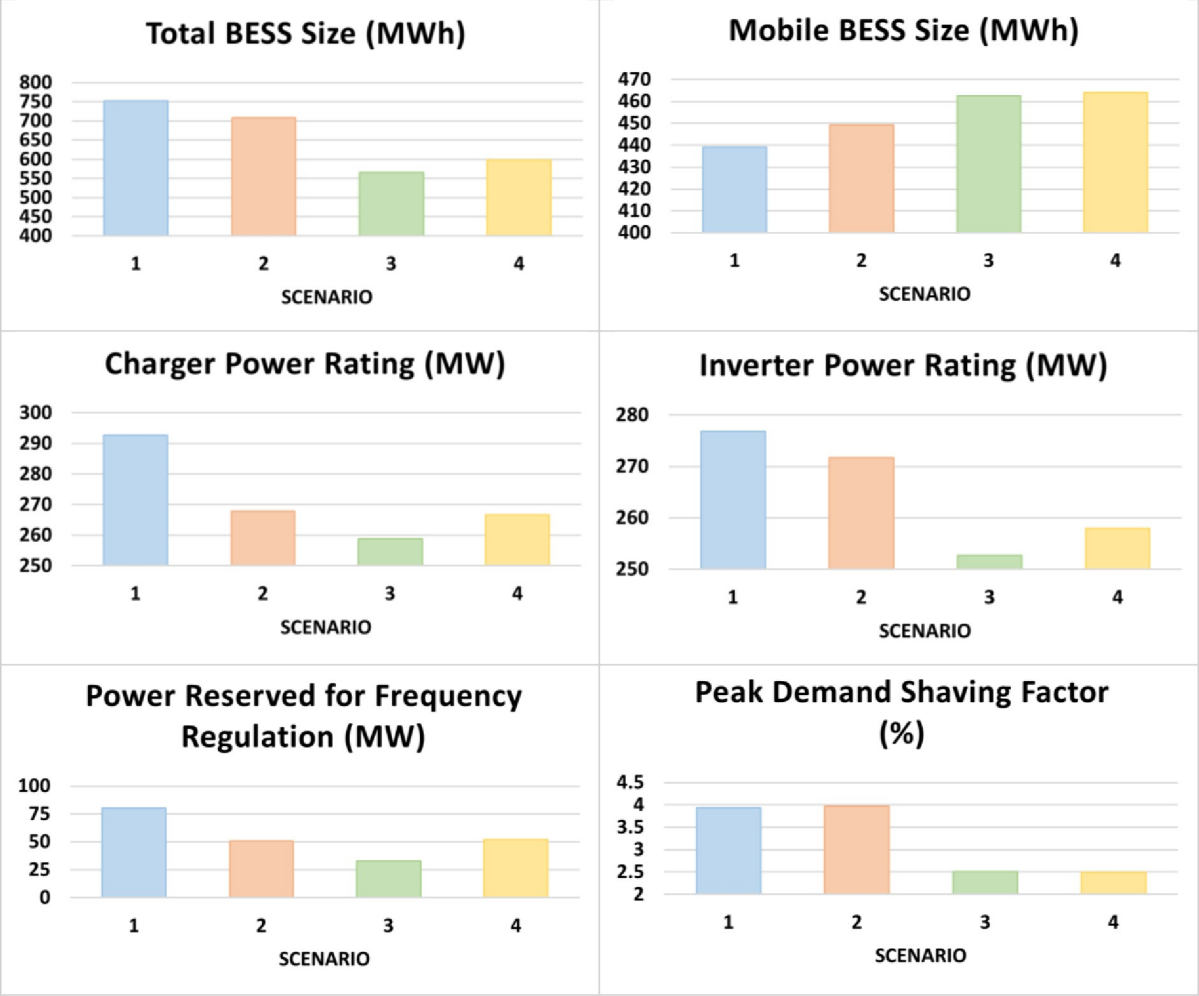
Optimized sets of the six control variables in four different scenarios (1, 2, 3, 4) under the DC priority operation mode.

**Fig 9:**
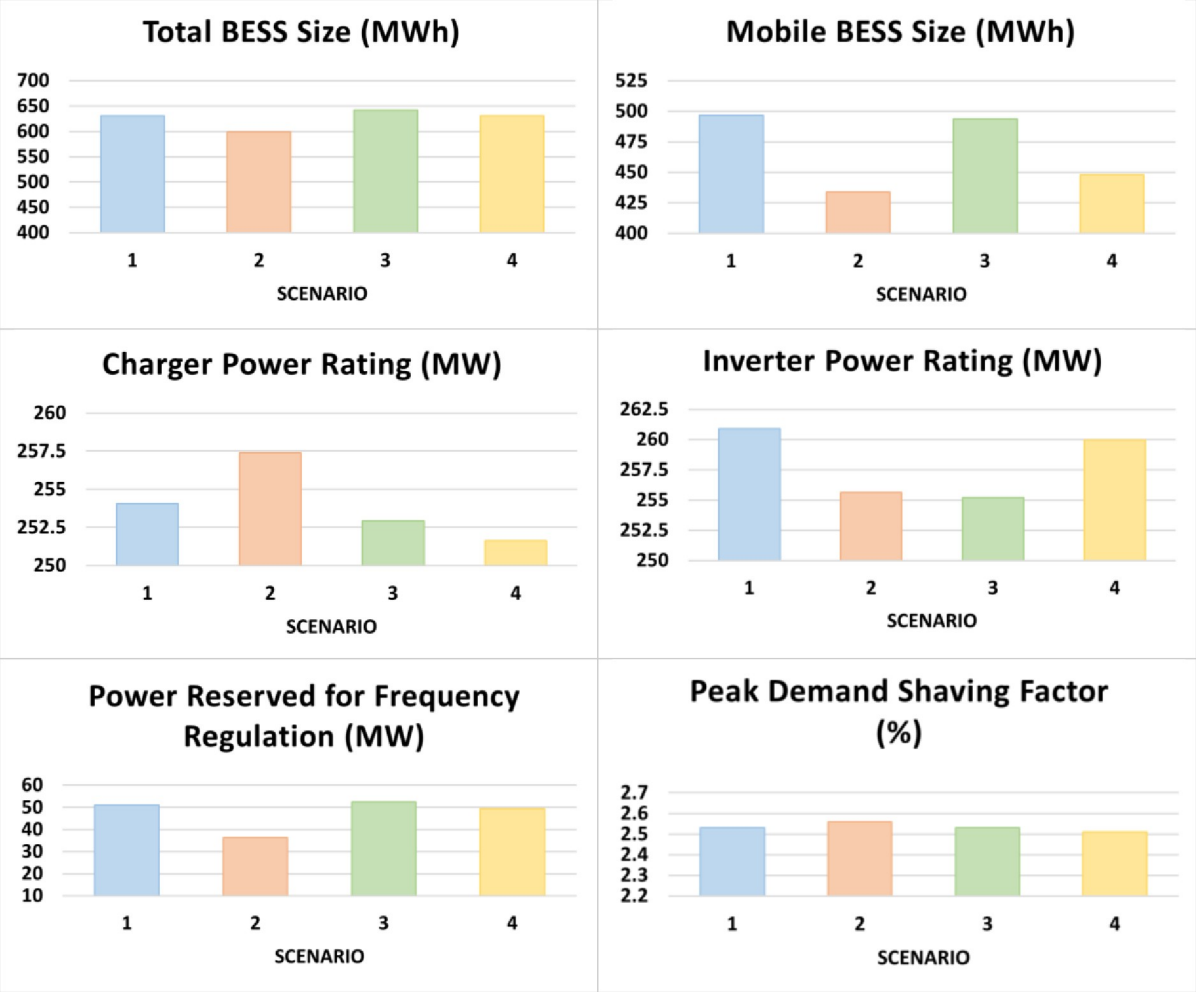
Optimized sets of the six control variables in four different scenarios (1, 2, 3, 4) under the AC priority operation mode.

**Fig 10:**
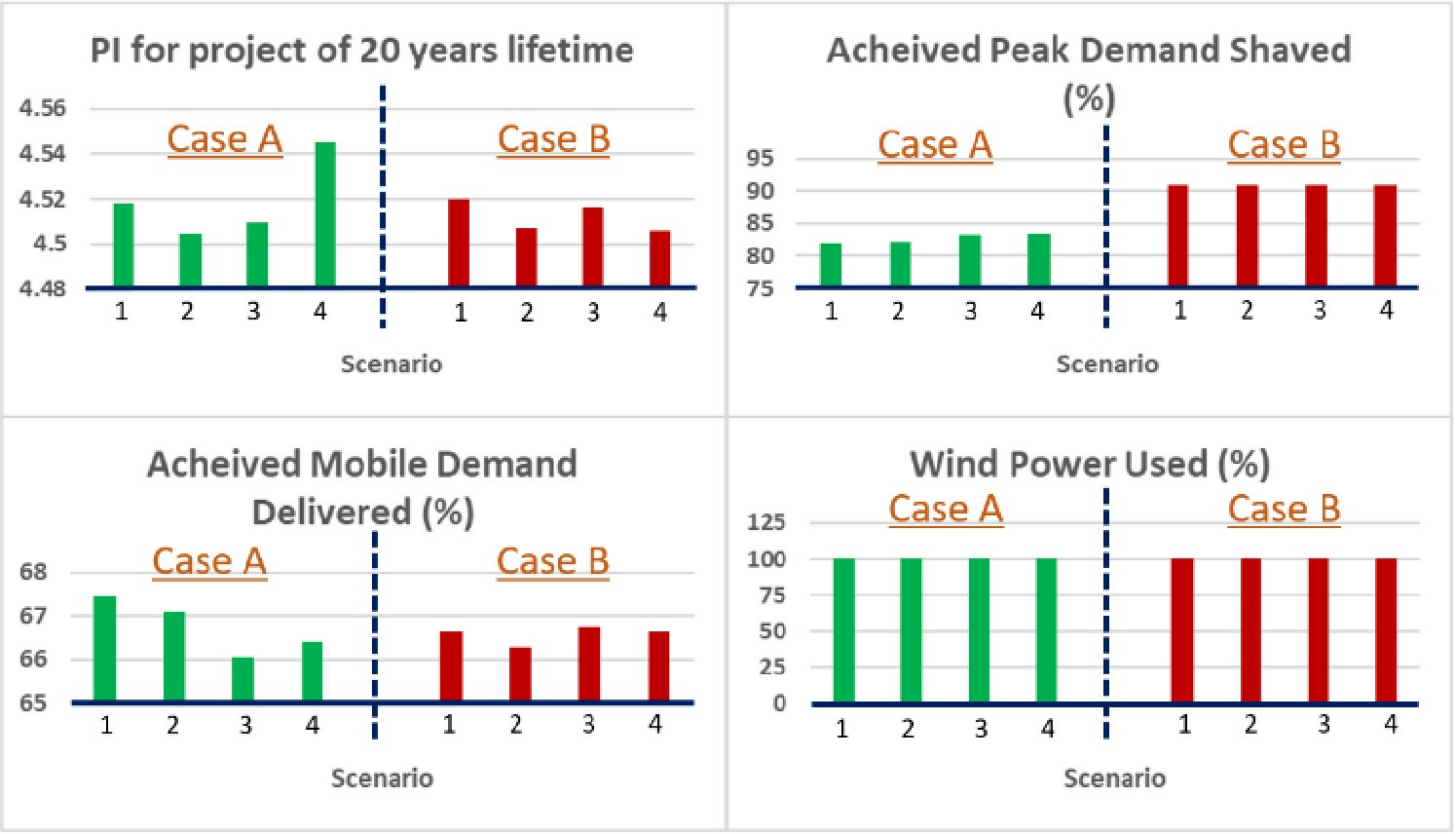
Four BESS performance indices (PI and Indices 2–4) with the optimized control variables in the four scenarios with the DC priority (case A) and the AC priority (case B).

In this subsection, the optimization results of the six control variables in four scenarios are presented. Each scenario had a different weight vector. Scenario 1: ***w*** = [0.25, 0.25, 0.25, 0.25], Scenario 2: ***w*** = [0.3, 0.15, 0.25, 0.3], Scenario 3: ***w*** = [0.5, 0.15, 0.15, 0.2], Scenario 4: ***w*** = [0.7, 0.1, 0.1, 0.1]. Under each scenario, results are obtained for the two operation modes: DC priority which indicates supplying the EVCS networks via the mobile BESS, as shown in Fig 8; and AC priority which indicates supplying the ECA demand via the stationary BESS, as shown in Fig 9. One of the key differences between the two modes/cases is that, with more focus on IA (Scenario 4), DC priority leads to a larger mobile BESS size, which is not the case of AC priority. The four performance indices are presented in Fig 10. For the data used, in all cases, Index 4 reaches or is very close to 100%, while there is more actual peak demand shaved in the AC priority mode than the DC priority mode.

On average, the PI at the end of the project lifetime is slightly higher under the DC priority than in the AC priority. However, all PIs are in the range of 4.4 ~ 4.6, which is equivalent to a 7~8% annual return over the 20-year project lifetime. Without a mobile application, we re-ran the model in the User Mode and calculated the PI values at the end of the project lifetime. The results are presented in Table 3. If the BESS has only one application (ECA), the PI in most cases is below 1, which means the project cannot break even. Adding frequency regulation as a secondary application approximately doubles the PI, but, in the best case, it only corresponds to an annual return of 3%. It is obvious that the combined stationary and mobile BESS applications, conceptualized in Figs 2 and 3, can increase the profitability and therefore the IA of the larger-scale deployment of BESS.

**Table 3:** PIs of different application combinations: 3 applications (EVCS, FR, ECA), 2 applications (FR, ECA), and 1 application (ECA only).

**Scenario**	**Profitability Index (PI) under the DC Priority**		
	*3 Applications*	*2 Applications*	*1 Application*
**1**	4.5180	1.7473	0.9388
**2**	4.5048	1.5905	1.0477
**3**	4.5095	1.0059	0.5969
**4**	4.5455	1.1677	0.5416

**Scenario**	**Profitability Index (PI) under the AC Priority**		
	*3 Applications*	*2 Applications*	*1 Application*
**1**	4.52	1.1258	0.5278
**2**	4.5069	1.0208	0.5803
**3**	4.5163	1.1337	0.5218
**4**	4.5062	1.1012	0.5214

### 3.2 Multi-objective optimization

This subsection presents the effort to perform multi-objective optimization of the proposed BESS design. The aim here is to obtain the profile of the Pareto frontier, i.e., the set of optimized solutions that cannot improve an objective without degrading at least one other objective. As mentioned earlier, the focus is on Index 2 and Index 3, the performance indicators for the DC and AC demands met by the BESS. Given the overall size of the BESS, it is not difficult to see that the two indices related to the portions of mobile and stationary batteries, which are trending in opposite directions and cannot be improved at the same time. It is of practical interests to see the trade-off.

**Fig 11:**
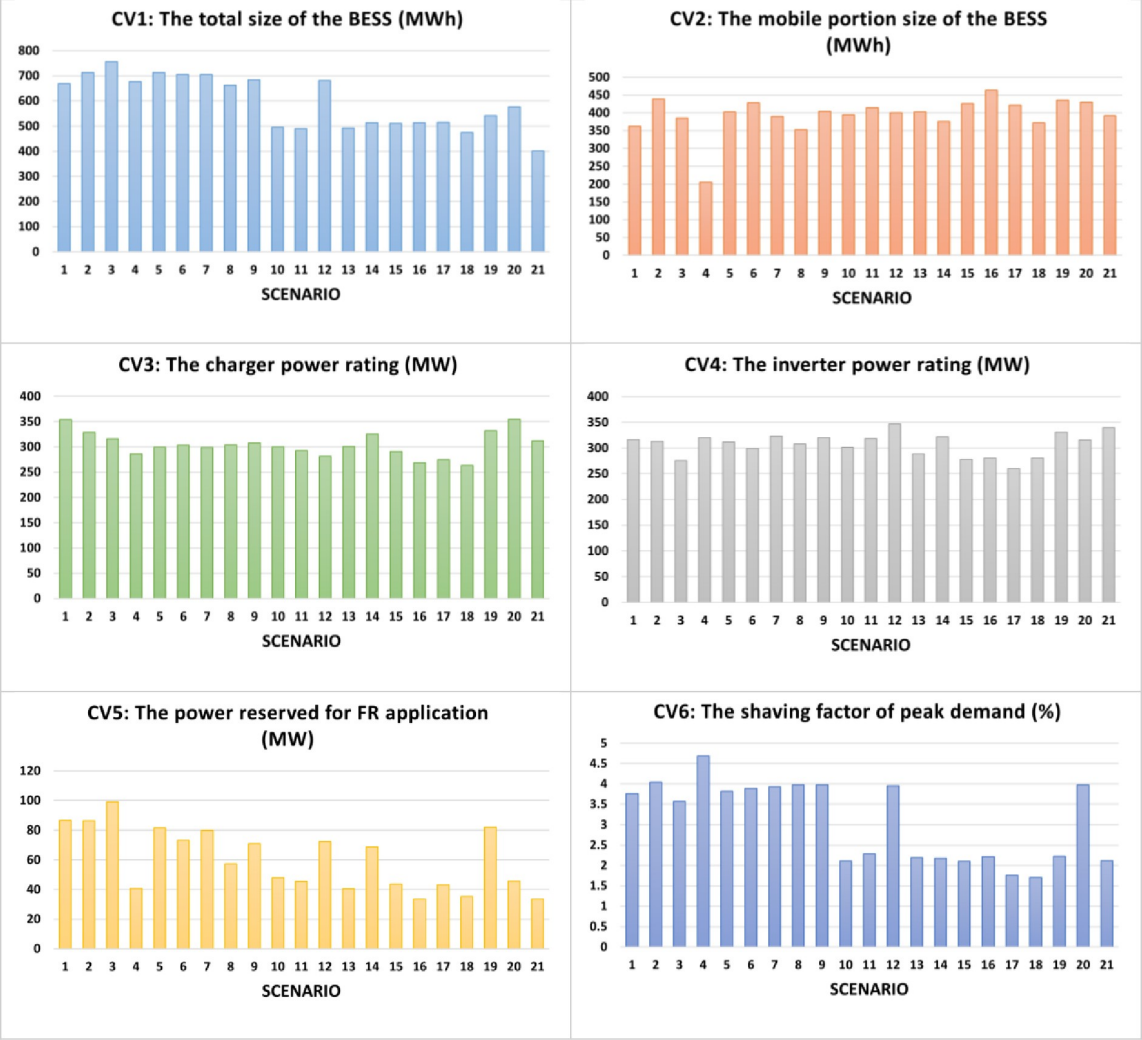
Optimal solutions of the six control variables with 21 weight factors from 0 (Scenario 1) to 1 (Scenario 21) in step of 0.05 under DC priority mode.

**Fig 12:**
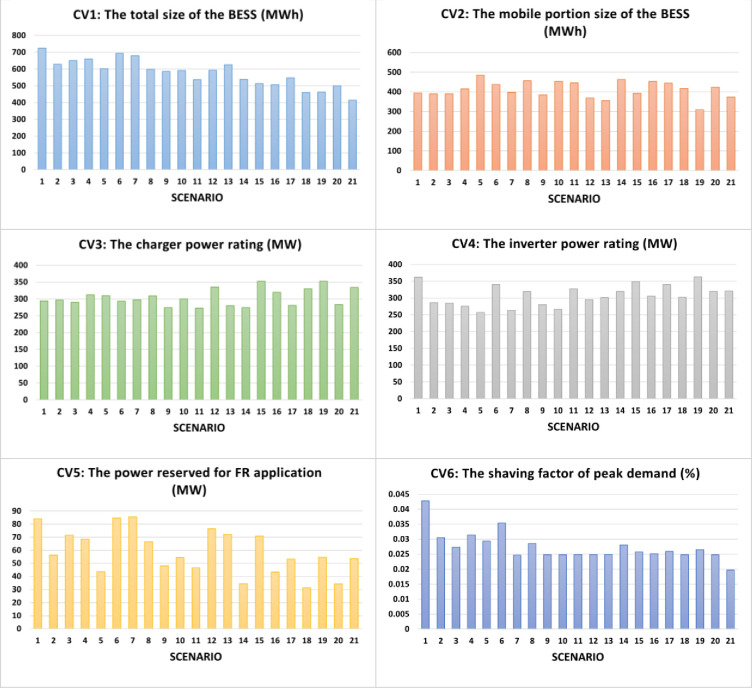
Optimal solutions of the six control variables with 21 weight factors from 0 (Scenario 1) to 1 (Scenario 21) in step of 0.05 under AC priority mode.

**Fig 13:**
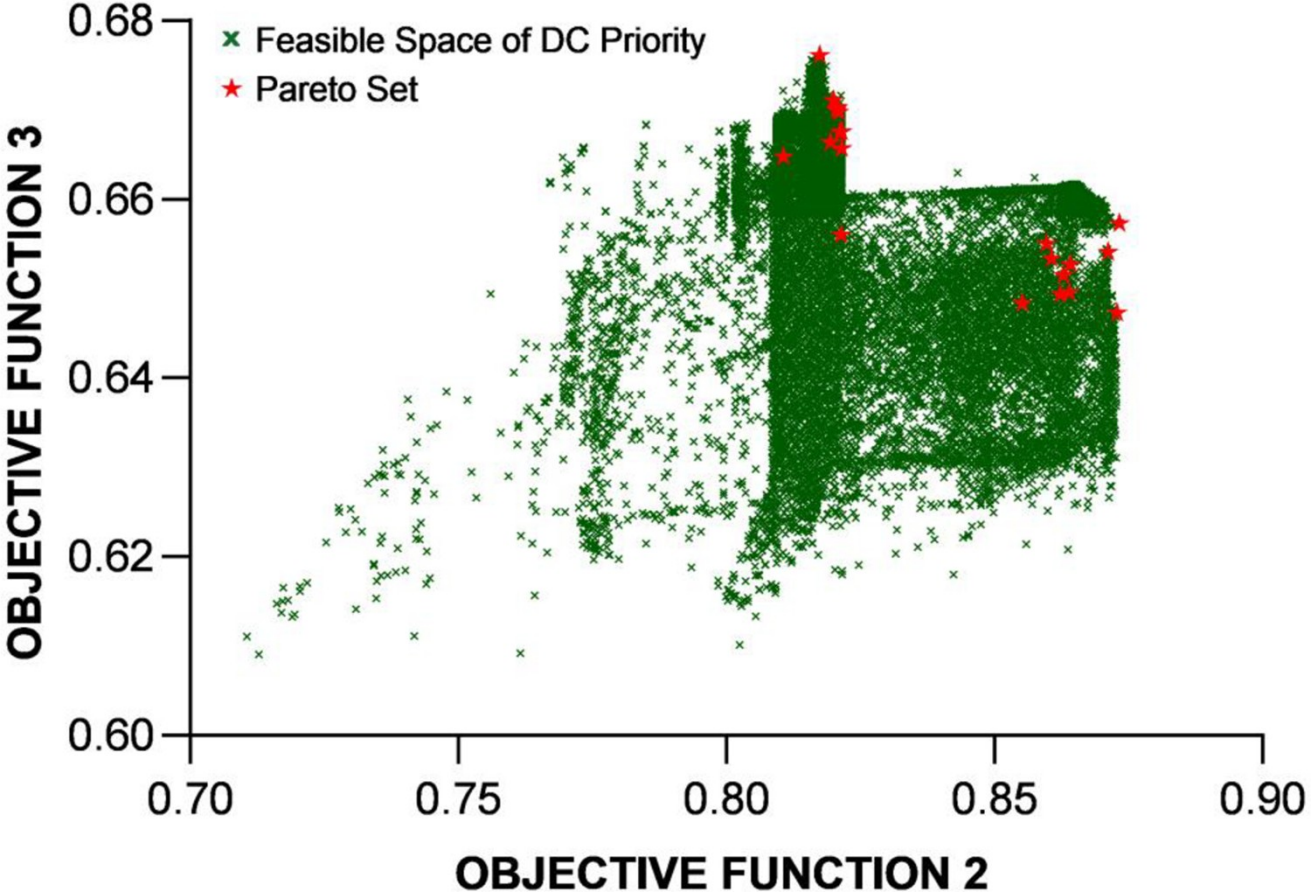
The profile of feasible solutions under the 21 scenarios and the Pareto set for the two objective functions under the DC priority operation mode.

**Fig 14:**
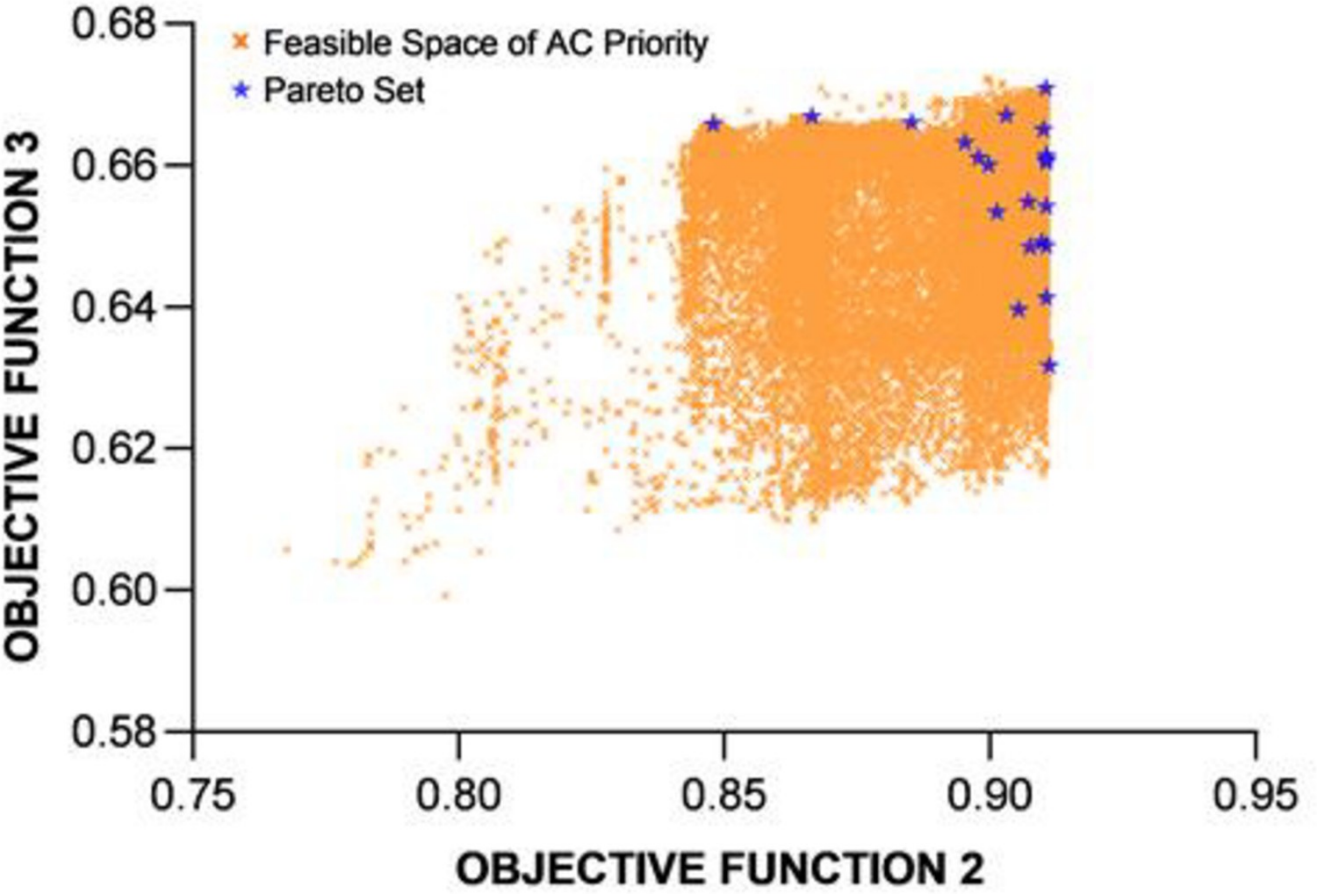
The profile of feasible solutions under the 21 scenarios and the Pareto set for the two objective functions under the AC priority operation mode.

To start with, a reduced dimensional optimization problem needs to be formulated. The linear scalarizing method is used to solve multi-objective optimization via the definition of the following weighted average of the two objective functions:

$$Maximize\;F\prime =wf_{2}+(1-w)f_{3}$$ (17)

where *w* is a real number between 0 and 1. From the results in the previous subsection, in all cases, the optimum fitness *F* is reached when the value of the investment attractiveness (IA) equals or exceeds 0.85 (*f*_1_≥0.85) and the value of the actual percentage of wind energy utilized equals 100% (*f*_4_ = 1). These will be two additional constraints of the new optimization problem. Now, to obtain the Pareto set of optimum solutions, optimizations are conducted with *w* = [0.00, 0.05, 0.10,…,1.00] of for each operational mode (DC and AC). Figs 11 and 12 present the optimized values for the six control variables under the DC and AC priorities, respectively. Figs 13 and 14 depict the respective Pareto set of optimal solutions.

**Fig 15:**
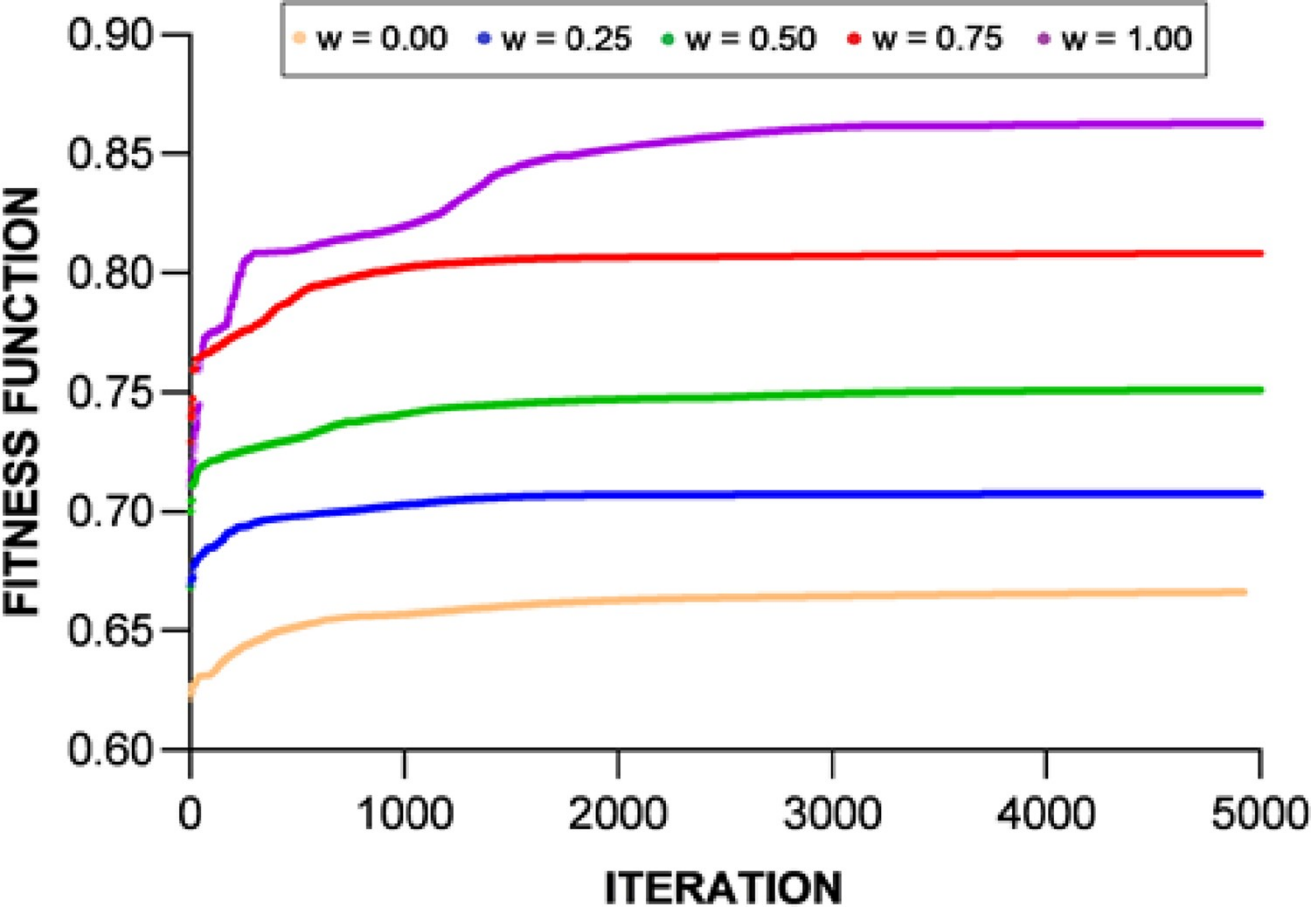
The convergence of the values of the fitness function in five scenarios under the DC priority operation mode.

**Fig 16:**
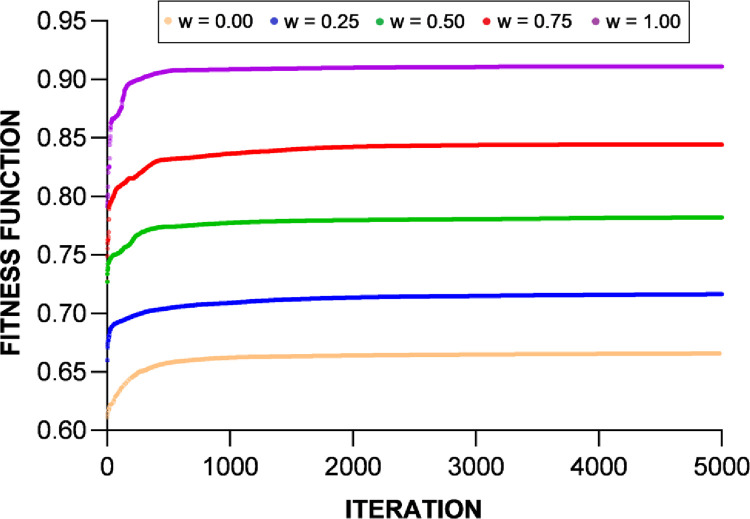
The convergence of the values of the fitness function in five scenarios under the AC priority operation mode.

To demonstrate the validity of the optimization results, the convergence plots of the new fitness function *F*’ are shown in Fig 15 for the DC priority operation mode and Fig 16 for the AC priority operation mode. The profiles of the Pareto sets in Figs 13 and 14 basically trace the upper-right boudary of the feasible region (which is the superposition of all the feasible solutions during the GA process) since both objectives are to be maximized. The Pareto frontier of the AC priority mode exhibits two lines (upper and right) along which small changes in one objective function come with large changes in the other. Interestingly, in the DC priority mode, the Pareto optimal solutions appear to be clustered in two regions, one with *f*_2_~0.86 and the other with *f*_2_~0.82. It is understandable that the former case corresponds to higher *f*_3_ since increasing the capability of meeting DC demand will reduce that of meeting AC demand. The cause of this phenomenon is subject to follow-up studies.

## 4. Conclusions

A multi-objective optimization framework is demonstrated for the design of a new form of BESS. Deployed with RES, the BESS comprises both conventional stationary batteries to meet intermittent demands from grid services and mobile batteries transported to meet continuous DC demand from EVCSs. The developed simulation model provides quantitative evidence that combining stationary and mobile applications can increase the profitability of BESS investment and reduce the curtailment of RES, while promoting electrical transportation.

Two operation priorities (DC and AC) and scenarios with different weights on performance indices are simulated and compared, which will be valuable for future demonstration project planning. In all simulation cases, the ratio of the actual power from the RES stored by the BESS to the total power available for storage according to the input data (Index 4) reached or was very close to 100%, while there was more actual peak demand shaved in the AC priority mode than the DC priority mode. Index 4 evaluated the performance of the BESS in maximizing the utilization of renewables that would be otherwise wasted.

On average, the profitability index at the end of the project lifetime was slightly higher under the DC priority than in the AC priority. However, all recorded PIs were in the range of 4.4 ~ 4.6, which is equivalent to a 7~8% annual return over the 20-year project lifetime. Without the mobile application, and with only one application (ECA), the PI in most cases was below 1, which means the project cannot break even and concludes that the combined stationary and mobile BESS applications can increase the profitability and therefore the investment attractiveness of the larger-scale deployment of BESS.

Further, Pareto frontier of a reduced-dimensional optimization problem is obtained to show the trade-off between two design objectives. This work lays the foundation for more in-depth studies such as improved multi-objective optimization techniques. Future work can also include the logistics systems modeling in the mobile BESS operation and financial analysis.

## Nomenclature

AC: Alternative Current
BESS: Battery Energy Storage System
CF: Cash Flow
CV: Control Variable
DC: Direct Current
ECA: End-Consumer Arbitrage
EF: Efficiency Factor
EIA: Energy
EV: Electric Vehicle
EVCS: Electric Vehicle Charging Station
FR: Frequency Regulation
GA: Genetic Algorithm
GHG: Greenhouse Gas
IA: Investment Attractiveness
OM: Operation and Maintenance
PI: Profitability Index
RES: Renewable Energy Source
SOC: State of Charge
UG: Utility Grid
VG: Value Generated
